# Postoperative cognitive dysfunction in elderly patients with colorectal cancer: A randomized controlled study comparing goal-directed and conventional fluid therapy

**DOI:** 10.1515/med-2024-0930

**Published:** 2024-03-27

**Authors:** Bin Wu, Yuanyuan Guo, Su Min, Qiuju Xiong, Lei Zou

**Affiliations:** Department of Anesthesiology, The First Affiliated Hospital of Chongqing Medical University, Youyi Road 1#, Yuzhong District, Chongqing 400016, People’s Republic of China; Department of Anesthesiology, The First Affiliated Hospital of Chongqing Medical University, Chongqing 400016, People’s Republic of China

**Keywords:** goal-directed fluid therapy, colorectal cancer, postoperative cognitive dysfunction, elderly patients

## Abstract

To investigate the impact of goal-directed fluid therapy (GDFT) on postoperative cognitive dysfunction (POCD) in elderly patients with colorectal cancer, we conducted a randomized controlled trial. Eighty elderly patients who underwent elective laparoscopic radical resection of colorectal cancer were randomly assigned to either the GDFT group or the conventional fluid therapy group. The primary outcome was the incidence of POCD during the initial 7 postoperative days, while secondary outcomes included inflammatory marker levels such as interleukin-6 (IL-6) and S100β protein, hemodynamics, level of lactic acid, postoperative functional recovery, and complications. Among 88 randomized patients, 80 were evaluable for the primary outcome. The incidence of POCD was significantly lower in the GDFT group (15.0%) compared to the conventional fluid therapy group (30.0%), with the highest occurrence observed on day 3 postoperatively in both groups (*P* < 0.05). IL-6 and S100β concentrations were consistently lower in the GDFT group than in the conventional fluid therapy group at the corresponding time points (*P* < 0.05). The GDFT group exhibited more stable perioperative hemodynamics and lower lactate levels (*P* < 0.05). Moreover, patients in the GDFT group exhibited better postoperative functional recovery indicators and a lower incidence of postoperative complications (*P* < 0.05). In summary, GDFT appears to reduce the incidence of early POCD, accelerate postoperative recovery, and enhance overall prognosis.

## 1 Introduction

Postoperative cognitive dysfunction (POCD) is a common central nervous system complication after anesthesia for surgery [1]. POCD refers to patients who show cognitive decline after anesthesia or surgery, including impaired learning and memory, personality changes, and anxiety [2]. According to the “Recommendations for the Nomenclature of Cognitive Change Associated with Anesthesia and Surgery-2018,” POCD is considered a type of perioperative neurocognitive disorder (PND) [3].

The aging population in China is increasing, and the incidence of colorectal tumors is high in the elderly population, resulting in considerable challenges in the management of perioperative anesthesia in elderly patients [4]. This population is more likely to suffer from postoperative complications and mortality. Among them, POCD is a common neurological complication in elderly patients who undergo surgery for colorectal cancer [5]. The occurrence of POCD can lead to complications, delayed recovery, prolonged hospitalization, increased hospitalization costs, and increased mortality, resulting in a significant burden for families and society [6]. Therefore, optimizing the perioperative management strategy to reduce the incidence of POCD has far-reaching significance for the postoperative recovery and prognosis of elderly patients with colorectal cancer.

Goal-directed fluid therapy (GDFT) is a new method of perioperative volume management that has developed rapidly in recent years and is currently widely used [7]. Evidence shows that GDFT is efficient in improving the prognosis of patients [8]. GDFT can effectively optimize the relationship between tissue perfusion and volume load in elderly patients, thereby improving postoperative efficacy, reducing complications, and promoting rapid postoperative recovery [9]. Several studies have indicated that intraoperative hemodynamic stability can be better maintained through GDFT to guide intraoperative volume management, thereby ensuring blood supply and oxygen supply to various important organs, reducing the systemic inflammatory response, and improving prognosis [10,11]. However, the protective effect of GDFT on postoperative cognitive function in elderly patients undergoing colon cancer surgery remains unclear.

Past studies suggest that the pathophysiological processes of POCD may involve various factors, among which interleukin-6 (IL-6) and S100β are considered effective biomarkers for POCD. IL-6, a pro-inflammatory cytokine, has been implicated in neuroinflammation and shown to be associated with cognitive impairment after surgery [12]. Similarly, S100β, a central nerve-specific protein, has been linked to cognitive impairment and neurocognitive deficits, particularly in the context of brain injury and postoperative complications [13,14]. Therefore, utilizing IL-6 and S100β may help elucidate the impact of GDFT on cognitive function.

In this study, we conducted a randomized controlled trial to analyze the impact of GDFT on intraoperative hemodynamics in elderly patients undergoing colon cancer surgery. Subsequently, we assessed differences between GDFT and the control group in terms of POCD incidence, postoperative recovery, and complications to comprehensively evaluate the role of GDFT in such patients.

## 2 Materials and methods

The study protocol was reviewed and approved by the Ethics Committee of The First Affiliated Hospital of Chongqing Medical University (2020-134), and all subjects and their families agreed to participate in this study and signed the informed consent.

### 2.1 Research subjects

Patients >60 years old who underwent elective laparoscopic radical resection of colorectal cancer between July 2020 and June 2021 in the Department of Gastrointestinal Surgery of The First Affiliated Hospital of Chongqing Medical University were selected. All patients’ consent forms were signed.

Patients were included if the following criteria are met: (1) colorectal cancer patients with preoperative pathological confirmation of colorectal cancer who required surgical treatment; (2) age >60 years old; (3) physical fitness status score (ECOG/WHO/Zubrod score) ≥2 points; (4) American Society of Anesthesiologists (ASA) physical classification I–III; (5) New York Heart Association cardiac function classification I–II; and (6) preoperative hematocrit >35%.

Patients were not included if any of the following conditions are met: (1) preoperative complications of severe cardiac insufficiency (cardiac index [CI] ≤2.2 l/min/m^2^); (2) history of mental illness or neurological disease; (3) use of drugs that may affect cognitive function; (4) patients with hepatic or renal insufficiency: Severe hepatic dysfunction (Child Turcotte Pugh grade C) or renal impairment (creatinine higher than the upper limit of normal); (5) patients with preoperative infection, autoimmune disease, active hepatitis, or blood system disease; (6) patients who received radiotherapy and chemotherapy before surgery; (7) Montreal cognitive assessment (MoCA) scale score ≥26 points (for subjects with <12 years of education, 1 point was added to the score); (8) operation time <2 or >6 h; (9) patients who received blood product infusion during the perioperative period; and (10) patients who have failed to extubate their endotracheal tube after surgery.

### 2.2 Study design and intervention

This study was a prospective randomized controlled trial. In brief, a research assistant, not part of the trial, used www.randomization.com to randomly assign subjects (1:1 ratio) to the experimental group (group G) or control group (group C). Group G followed GDFT volume management, while group C followed conventional fluid replacement. The research assistant, responsible for randomization, sealed group information in envelopes. On surgery day, envelopes were opened, and subjects were grouped by number. Throughout the study, all involved personnel, except the anesthesiologist, remained blinded to patient grouping.

Sample size was calculated by: PASS 15.0 statistical software. The ratio of the experimental group to the control group was set to 1:1, power = 0.80, *α* = 0.05, and *β* = 0.20. The incidence of POCD was estimated to be 30.0% in elderly patients who undergo surgery for colorectal cancer according to the literature, and the incidence of POCD was estimated to be 6.0% in GDFT group according to our pre-test, and the total sample size was calculated is 72 cases (36 cases in each group). Assuming a dropout rate of 10%, the total sample size was 80 cases (40 cases in each group).

Upon the patient’s arrival to the operating room, an intravenous infusion channel was established and radial artery puncture and catheterization were performed to continuously monitor arterial blood pressure. The multifunctional monitor was used to continuously monitor heart rate (HR), mean arterial pressure (MAP), and pulse oxygen saturation (SpO_2_). The Narcotrend monitor was used to monitor sedation in patients. After the arterial puncture, the FloTrac/Vigileo device was connected for continuous monitoring of CI, stroke volume (SV), and stroke volume variation (SVV). Anesthesia was induced with midazolam (0.05 mg/kg), propofol (1–2 mg/kg), sufentanil (0.4 µg/kg), and vecuronium (0.1 mg/kg) via intravenous injection. Intraoperative anesthesia was a combination of intravenous propofol (3 mg/(kg h)), remifentanil (0.01 mg/(kg h)), and inhaled sevoflurane (1–3%). Sedation depth (Narcotrend index maintained between 40 and 60) and muscle relaxation with intermittent vecuronium bromide (0.05 mg/kg) were adjusted based on surgical requirements.

During the operation, the following values were maintained in the two groups: SpO_2_ >95%, hemoglobin (Hb) >80 g/l, core body temperature >37°C, HR 50–100 beats/min, MAP 60–100 mm Hg, and intraoperative CO_2_ pneumoperitoneum pressure 10 cm H_2_O.

The control group underwent fluid therapy according to the conventional guidelines: total amount of fluid input = physiological requirement + cumulative amount of fluid loss + amount of body fluid loss in the third interval + amount of continued fluid loss. Physiological requirements and cumulative fluid deficits were calculated according to the “4:2:1” rule as follows: for the first 10 kg, the deficit is calculated as 4 ml/(kg h), for the second 10 kg, it is 2 ml/(kg h), and for the third 10 kg and later, it is 1 ml/(kg h). The fluid replacement regimen consisted of the physiological requirement and cumulative loss (crystalloid) + compensatory expansion (5 ml/kg, colloid) + the amount of body fluid loss in the third interval (3 ml/kg/h, crystalloid) + the amount of continued fluid loss. The crystalloid solution was lactic acid Naringer’s solution, and the colloid solution was hydroxyethyl starch 130/0.4 sodium chloride injection. The scheme is shown in Figure S1.

The experimental group underwent capacity management according to the GDFT process. During the operation, the patients were continuously instilled with crystalloid at 8 ml/(kg h) to maintain the basal fluid volume. Under the conditions of general anesthesia and mechanical ventilation, the FloTrac/Vigileo system monitor was connected to closely monitor the hemodynamic parameters. The intraoperative SVV was used as the guiding index. When the SVV exceeded 13%, a fluid shock test was performed in which 200 ml of colloid were administered to the patient; the infusion was completed within 5 min, and the changes in SVV were observed and recorded. When the measured SVV was >13% (for 5 min), another 200 ml of colloid was administered until the SVV was ≤13%. The crystalloids were rehydrated with lactic acid Naringer’s solution, and the colloids were refilled with a hydroxyethyl starch 130/0.4 sodium chloride injection. The scheme is shown in Figure S2.

Regarding postoperative analgesia, all patients received patient-controlled intravenous analgesia (PCIA). For the drug formulation, tramadol 800 mg and flurbiprofen axetil 200 mg were diluted to a volume of 100 ml with 0.9% normal saline. The PCIA settings were 5 ml loading dose, 2 ml/h maintenance dose, and 15 min PCIA lock.

### 2.3 Primary outcome

According to the previous literature [15], the MoCA scale was used to assess the occurrence of POCD. The MoCA scale includes seven aspects: visuospatial and executive ability, naming ability, attention ability, language fluency, memory ability, abstract thinking ability, and orientation ability. The scores for each cognitive domain were counted separately, and the final total score was calculated. Because the scale is related to the educational level, 1 point was added to the score for individuals who completed <12 years of education. MoCA scores were determined in patients 1 day before surgery (Pre-D), 1 day after surgery (POD1), 3 days after surgery (POD3), and 7 days after surgery (POD7), and the test scores for each item were calculated. MoCA scores were assessed by the same professionally trained physician in a double-blind manner regarding grouping information.

The scores for each item were obtained before and after surgery for all patients; the scores before surgery were used as controls, and the postoperative test values were compared with the preoperative test values. If the postoperative score decreased by ≥20% compared with the preoperative score, it was considered postoperative functional deterioration. The patient was considered to have POCD if there was functional deterioration in ≥2 test items postoperatively.

### 2.4 Secondary outcome

#### 2.4.1 Inflammatory response indicators

Serum IL-6 and S100β levels were determined using 3 ml of venous blood collected from patients at Pre-D, POD1, POD3, and POD7. An enzyme-linked immunosorbent assay (ELISA) (Shenzhen Jingmei Bioengineering, 103963) was employed for IL-6 and S100β determination, following the manufacturer's instructions. The double-antibody sandwich ABC-ELISA method was utilized, involving monoclonal antibodies against human IL-6 or S100β, biotin-containing antibodies, streptavidin, and *o*-phenylenediamine. The concentration of IL-6 or S100β in the sample was calculated based on the standard curve.

#### 2.4.2 Hemodynamic indicators and lactic acid

Hemodynamic indicators (MAP, HR, CI, SV, and SVV) were continuously monitored using Flotrac/Vigileo in both the experimental and control groups at five time points: 5 min before anesthesia induction (T0), 5 min after induction (T1), the start of surgery (T2), 1 h at the beginning of surgery (T3), and the surgery's end (T4). At these time points, 0.5 ml of radial artery blood was collected from each patient to measure arterial lactic acid content using a blood gas analyzer.

#### 2.4.3 Postoperative recovery and complications

Postoperative management, overseen by physicians from the same medical group, involved monitoring various aspects. This included assessing recovery indicators such as the first exhaust time, initial liquid food intake, first ambulation time, and the overall duration of postoperative hospital stay. Additionally, postoperative complications within the first 7 days were meticulously recorded, encompassing issues like postoperative delirium, nausea and vomiting, fever, surgical site infection, heart failure, pulmonary infection, pulmonary edema, and hypoxemia. The entire postoperative hospital stay duration was also taken into account.

#### 2.4.4 Statistical analysis

Experimental data were analyzed using SPSS 22.0 software. In this study, intention-to-treat analysis was employed due to the high compliance of patients with GDFT treatment or conventional fluid therapy. Normality of measurement data was assessed with the Shapiro–Wilk test. Normally distributed measurement data were presented as mean ± standard deviation (*x* ± *s*). For comparison between two groups, the Student’s *t*-test was used for numerical variables and the Pearson’s chi-square test for categorical ones. A two-way analysis of variance followed by Šídák's multiple comparisons test was used in cases where more than two groups were compared with repeated measures. All statistical tests were two-sided, and *P* < 0.05 was considered statistically significant.

## 3 Results

**Figure 1:**
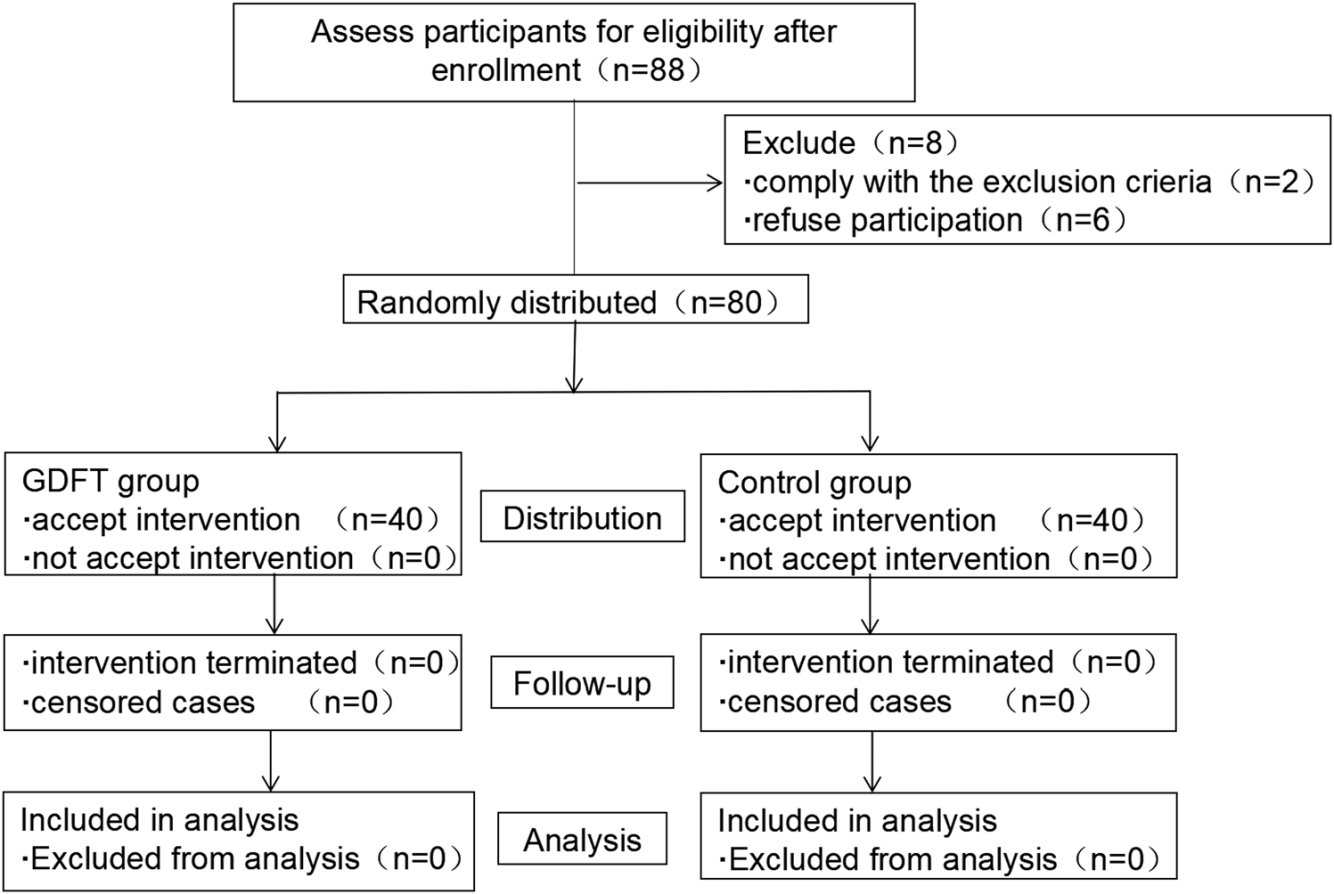
Flowchart of the included subjects. Abbreviation: GDFT, goal-directed fluid therapy.

A total of 88 patients who underwent elective laparoscopic radical resection of colorectal cancer were included in this study between July 2020 and June 2021. Among them, 2 patients were excluded because of preoperative complications including severe hepatic and renal insufficiency, 3 patients and their families refused to participate, and 3 patients refused MoCA test. Finally, 80 patients (40 in each group) completed the study and were included in the statistical analysis (Figure 1).

Table 1 shows the patient demographic data. There were no significant between-group differences in sex ratio, age, body mass index, years of education, ASA health status classification, cancer type, tumor node metastasis stage, anesthesia time, and operation time.

**Table 1:** Study characteristics of participants

Indicators	Group G	Group C	*P* value
Sex ratio (male/female)	23/17	21/19	0.372
Age (years)	66.37 ± 4.85	64.87 ± 5.12	0.182
BMI (kg/m^2^)	22.58 ± 1.61	23.58 ± 1.49	0.508
Years of education (years)	7.45 ± 3.61	7.75 ± 3.29	0.698
ASA health status classification (*n*, II/III)	22/18	19/21	0.365
Colon/rectal cancer (*n*)	25/15	27/13	0.468
TNM stage (*n*, %)			
Stage I	13 (32.5%)	11 (27.5%)	0.632
Stage II	17 (42.5%)	18 (45.0%)	0.914
Stage III	10 (25.0%)	11 (27.5%)	0.632
Anesthesia time (min)	153.6 ± 35.2	161.2 ± 33.8	0.328
Operation time (min)	128.4 ± 29.5	130.3 ± 25.9	0.760

Abbreviation: BMI, body mass index; ASA, American Society of Anesthesiologists; TNM, tumor node metastasis.

Before and during the surgery, no significant differences were observed in preoperative assessments, including vital signs and laboratory tests, between the two groups (Table S1). Group G had significantly lower total infusion volume than group C (*P* < 0.001). Group G also showed lower crystalloid infusion but higher colloid infusion compared to the control group (*P* < 0.001). Intraoperative urine volume and blood loss did not differ significantly between the groups (Table S2).

The MoCA scores on postoperative days 1, 3, and 7 were lower than the preoperative baseline in both groups, with the most notable decrease in day 3. While scores gradually recovered by day 7, they remained below the preoperative baseline. Group G exhibited higher MoCA scores compared to group C at the same time points, with a statistically significant difference (*P* < 0.05, Table S3).

POCD cases were identified based on MoCA scores, with 18 out of 80 patients (22.5%) experiencing POCD. The highest incidence occurred on postoperative day 3 in both groups. The GDFT group had six cases, resulting in a 15.0% POCD incidence, while group C had 12 cases, yielding a 30.0% incidence. The GDFT group demonstrated a significantly lower incidence of POCD compared to the control group (Table 2).

**Table 2:** The incidence of POCD in the two groups of patients

Incidence of POCD	Group G	Group C
The overall incidence (*n*, %)	6 (15.0)	12 (30.0)*
Incidence of POD_1_ (*n*, %)	2 (5.0)	3 (7.5)
Incidence of POD_3_ (*n*, %)	3 (7.5)	7 (17.5)
Incidence of POD_7_ (*n*, %)	1 (2.5)	2 (5.0)

Abbreviation: POCD, postoperative cognitive dysfunction; POD, day after surgery.

*Significant difference between group G and group C (*P* < 0.05).

The plasma levels of IL-6 and S100β were significantly elevated on postoperative days 1, 3, and 7 compared to preoperative baseline values in both groups (*P* < 0.001). The peak levels occurred on day 3 post-surgery, followed by a subsequent decrease. In the GDFT group, plasma IL-6 and S100β levels were significantly lower than those in the control group on postoperative days 1, 3, and 7 (*P* < 0.001, Table 3).

**Table 3:** Plasma IL-6 and S100β of patients at different time points

Indicators	Time points	Group G	Group C	*P* value
IL-6 (pg/ml)	Pre-D	51.65 ± 8.58	49.98 ± 10.15	0.882
	POD_1_	70.55 ± 8.92	79.53 ± 9.18*#	<0.001
	POD_3_	93.56 ± 9.12	102.32 ± 11.57*#	<0.001
	POD_7_	60.11 ± 6.49	65.91 ± 8.14*#	0.019
S100β (pg/ml)	Pre-D	138.43 ± 8.82	139.31 ± 9.22	0.987
	POD_1_	177.78 ± 8.31	207.56 ± 9.62*#	<0.001
	POD_3_	199.68 ± 9.71	233.11 ± 10.67*#	<0.001
	POD_7_	154.5 ± 6.46	176.59 ± 7.84*#	<0.001

Abbreviation: IL-6, interleukin-6; POD, day after surgery.

*Significant difference between group G and group C.

#Significant difference compared with Pre-D.

In the GDFT group, MAP decreased at T1, T2, and T3, but increased at T4. The control group exhibited a decrease in MAP at T1, T2, T3, and T4. At T3 and T4, the MAP in the control group was higher than that in the GDFT group. HR in the GDFT group decreased at T1, T2, T3, and T4 compared to T0, while the control group's HR decreased at T1, T2, and T3 and increased at T4. At T4, the control group had a higher HR than the GDFT group. CI in the GDFT group significantly decreased at T1 but increased at T2, T3, and T4. In the control group, CI decreased at all four time points. While no significant difference in CI was observed between the two groups at T1 and T2, the control group exhibited significantly lower CI than the GDFT group at T3 and T4. SV showed no significant difference between the two groups at T1 and T2, but at T3 and T4, the control group had significantly lower SV than the GDFT group. In the GDFT group, SVV did not exceed 13% at any monitoring point during the operation. Group C showed higher intraoperative SVV with an increasing trend. There was no significant difference between the two groups after anesthesia induction at T1. However, at T2, T3, and T4, SVV in group G was significantly lower than in group C. Average intraoperative lactic acid in group G gradually changed without a significant peak, while in group C, lactic acid increased 1 h after and at the end of the operation. No significant baseline differences in lactic acid were observed between groups G and C before anesthesia induction. After induction and at the beginning of surgery, lactic acid levels did not significantly differ between the two groups. However, 1 h and at the end of the surgery, lactic acid concentration was significantly lower in group G compared to group C (Figure S3).

Comparison of the postoperative intestinal ventilation time, liquid feeding time, and getting out of bed time between the two groups showed that the recovery time of group G was shorter than that of group C (*P* < 0.05), but there was no significant difference in postoperative hospitalization time between the two groups (*P* > 0.05, Table 4). Group G had a postoperative complication rate of 27.5%, whereas group C exhibited a higher complication rate of 52.5%. The incidence of postoperative complications was significantly lower in group G than in group C (*P* < 0.05, Table S4).

**Table 4:** Comparison of postoperative functional recovery and postoperative hospital stay of patients

Indicators	Group G	Group C	*P* value
Intestinal ventilation time (h)	18.5 ± 5.8	23.5 ± 6.2	<0.001*
Liquid feeding time (h)	50.5 ± 10.2	59.5 ± 11.5	<0.001*
Time to get out of bed (h)	15.2 ± 4.1	19.7 ± 4.5	<0.001*
Postoperative hospital stay (days)	6.2 ± 1.8	6.4 ± 1.9	0.630

*Significant difference between group G and group C.

## 4 Discussion

This study compared the use of GDFT with conventional fluid therapy in elderly patients who underwent colorectal cancer surgery. We aimed to explore the effect of GDFT on POCD in elderly patients with colorectal cancer, as well as the correlation between inflammatory responses and POCD, and to determine the role of GDFT in the prognosis of patients.

Preventing POCD constitutes a multifaceted and intricate challenge necessitating a comprehensive approach. Factors to be considered include anesthetic methods [16], perioperative pharmacological interventions [17], pain management [18], and the regulation of physiological parameters during surgery [19]. Liquid therapy, also known as fluid therapy, plays a crucial role in the surgical process. The perioperative fluid management is essential for maintaining adequate hydration, electrolyte balance, hemodynamic stability, and promoting optimal organ function during surgery [20]. The brain is highly sensitive to changes in fluid balance, even mild dehydration can lead to impairments in attention, memory, and executive function, as well as alterations in brain activity [21]. Therefore, fluid therapy plays a crucial role in preventing and treating POCD, supporting optimal brain function. Our investigation reveals that GDFT can alleviate the incidence of POCD in elderly surgical patients undergoing colon cancer surgery within the first 7 days postoperatively, significantly mitigating cognitive impairment. Importantly, our findings are consistent with earlier literature, reinforcing the validity of our results. A case–control study has shown that, for elderly patients with spinal stenosis, GDFT can mitigate short-term postoperative cognitive impairment [22]. Another randomized controlled trial also indicates that GDFT demonstrates a protective effect on cognitive function in patients undergoing radical esophageal cancer surgery [23]. This phenomenon has also been observed in another randomized controlled trial study on Hip Arthroplasty [24]. The above findings suggest that GDFT has a protective effect on postoperative cognitive function.

The mechanism underlying the development of POCD is not fully understood. Potential pathogenetic factors associated with POCD include neuroinflammation, surgical stress, hypoxia, hypotension, cerebral hypoperfusion, micro thrombosis, and temperature changes [25,26]. Neuroinflammation, in particular, has been identified as a key contributor to the development of POCD [27]. Studies have shown that inhibiting specific neuroinflammatory pathways, such as the NLRP3 signal, can ameliorate POCD [28]. In the present study, our research has uncovered that GDFT can mitigate postoperative levels of IL-6 and S100B in elderly cancer surgical patients. These two indicators have been previously identified in research as promising biomarkers for POCD. Several meta-analyses have underscored the potential role of inflammatory markers, revealing a significant association between IL-6 and the occurrence of POCD [29,30]. Other studies have also observed that, compared to non-POCD patients, individuals with POCD exhibit higher levels of S100β at postoperative 1 h, postoperative 2 days, and postoperative 9 days [31]. Therefore, our data, at the level of biomarkers, similarly support the protective effect of GDFT against POCD.

The mechanisms through which GDFT reduces the incidence of POCD and the levels of IL-6 and S100B may involve the hemodynamic stability and effective organ perfusion provided by GDFT. Our study demonstrates that patients undergoing GDFT exhibit greater intraoperative hemodynamic stability, coupled with lower lactate levels. In this study, we monitored intraoperative hemodynamic parameters, including MAP, HR, CI, SV, and SVV. The SVV of the GDFT group was significantly lower than that of the control group throughout. Similarly, both groups experienced a reduction in MAP and HR after anesthesia induction and at the start of surgery. However, in the GDFT group, MAP gradually returned to preoperative levels by the end of the operation, with no significant change in HR. In contrast, the control group consistently showed lower blood pressure than preoperative levels throughout the procedure. Those changes reflect the volume management scheme of GDFT, which can effectively reduce SVV and improve cardiac output. Moreover, maintaining stable hemodynamics is crucial for enhancing tissue and organ perfusion, ensuring optimal oxygen supply to the central nervous system and various organs [32]. In our study, we employed lactate as a sensitive indicator of human tissue perfusion [33] and found that, compared to the control group, GDFT significantly decreased lactate levels, thereby enhancing perfusion to tissues and organs.

In addition to mitigating POCD, our research has identified that GDFT can also reduce postoperative complications and promote postoperative functional recovery. This phenomenon has been widely documented in other research studies [34,35,36]. However, a recent multicenter study focusing on emergency abdominal surgery [37] revealed that, compared to the control group, GDFT did not exhibit significant differences in postoperative mortality, major complications, or hospital stay. This outcome suggests that GDFT may not confer benefits to all patients undergoing surgery, such as emergency procedures.

### 4.1 Limitation

The study possesses certain limitations. First, the definition of the elderly population in this research is set at 60 years old rather than the more commonly used 65 years. This decision is based on the consideration that, in developing countries, 60 years can be considered elderly [38], facilitating a broader investigation into the neuroprotective effects of GDFT in the elderly population. Second, the study primarily includes a Chinese population, potentially limiting the generalizability of the results to other populations. Third, the research does not observe the impact of GDFT on long-term PND, and the long-term cognitive function and postoperative outcomes associated with GDFT remain to be explored.

## 5 Conclusions

In elderly colorectal cancer surgical patients, GDFT seems to play a role in reducing the occurrence of early POCD, expediting postoperative recovery, and improving overall prognosis. The underlying mechanisms may be associated with maintaining stable hemodynamics, ensuring tissue and organ perfusion, and reducing neuroinflammation.

## Supplementary Material

supplementary material
