# Supplementary material for: Postoperative cognitive dysfunction in elderly patients with colorectal cancer: A randomized controlled study comparing goal-directed and conventional fluid therapy
Source: Open Med (Wars). 2024 Mar 27;19(1):20240930. doi: 10.1515/med-2024-0930 (PMC10997005; doi:10.1515/med-2024-0930)
Supplement: supplementary material [file med-2024-0930-sm.pdf]

Supplementary material

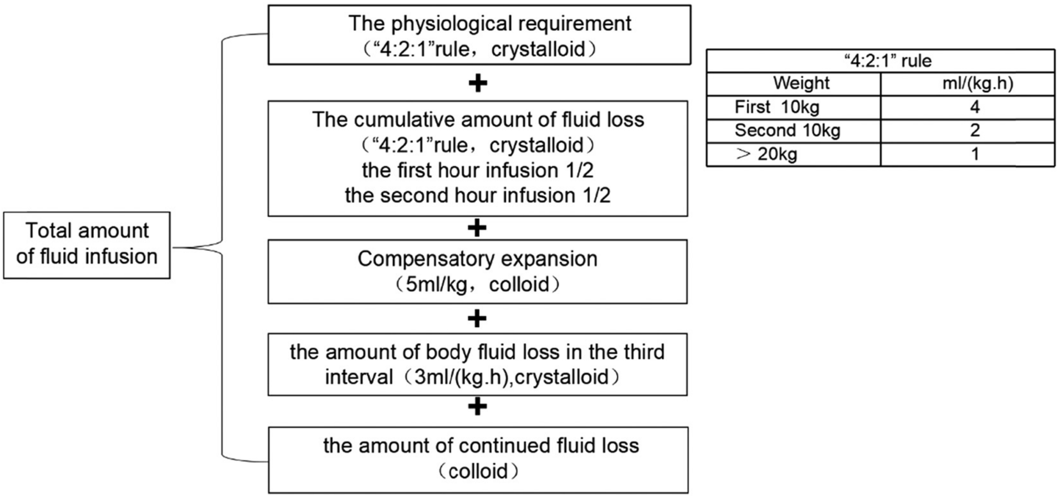

Figure S1: The scheme of conventional fluid therapy.

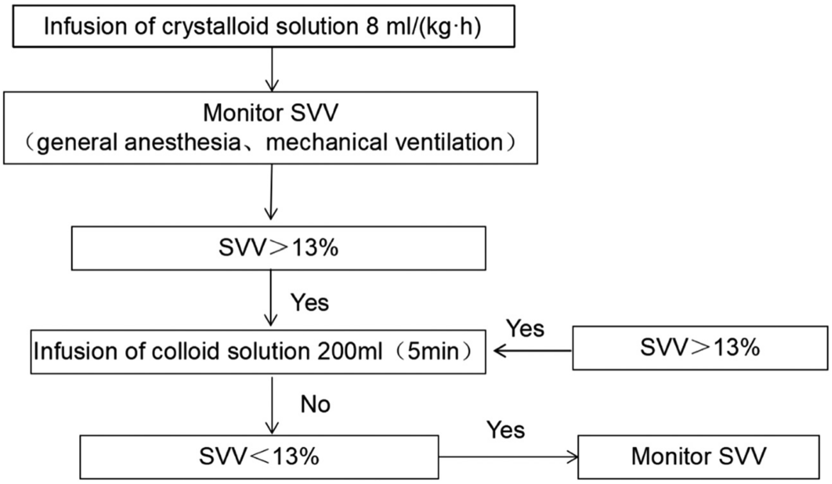

Figure S2: The scheme of goal-directed fluid therapy. Abbreviation: SVV, Stroke volume variation.

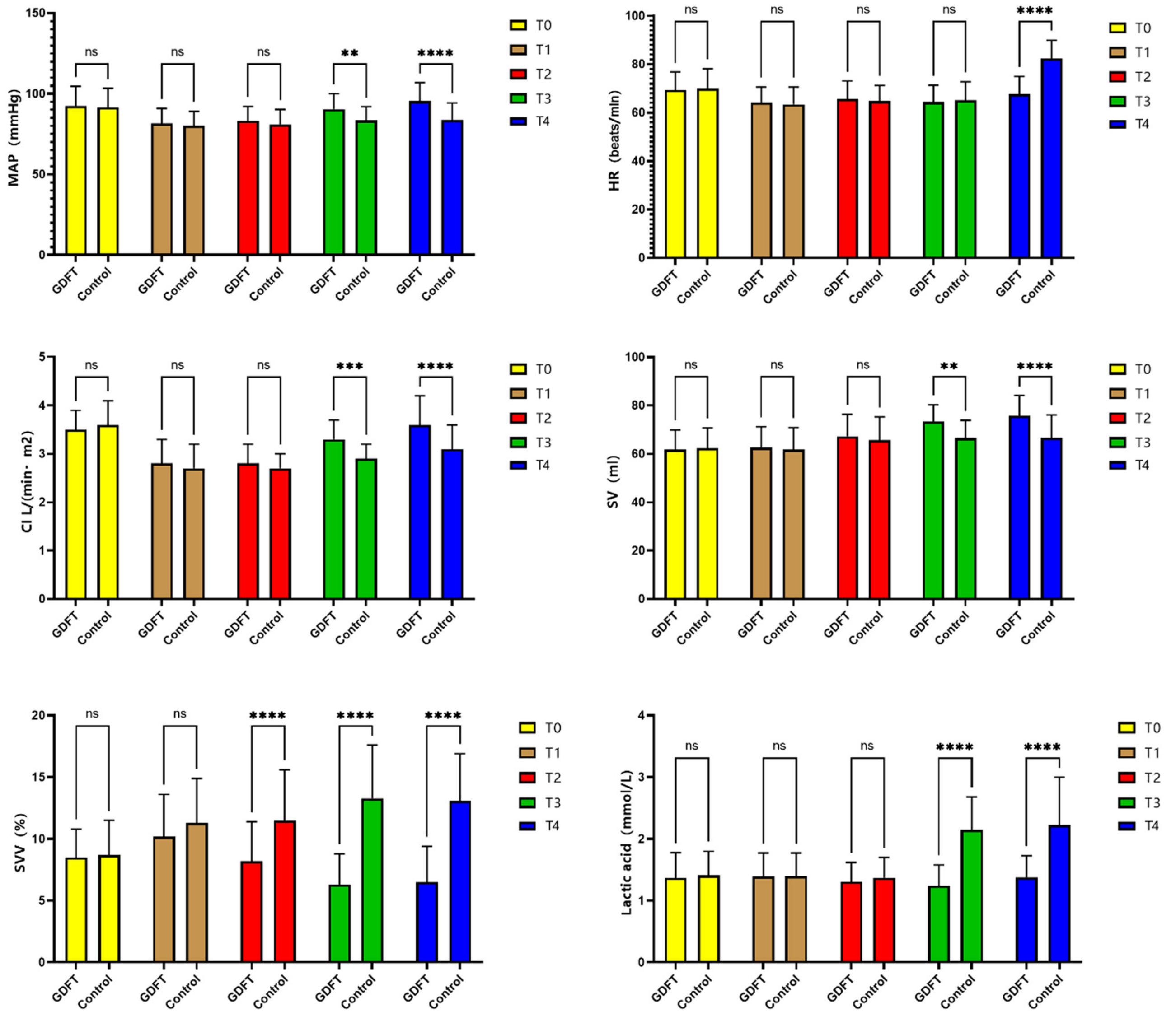

Figure S3: Comparison of hemodynamics and lactic acid of patients at different time points. Abbreviation: MAP, Mean arterial pressure; HR, Heart rate; CI, Cardiac index; SV, Stroke volume; SVV, Stroke volume variation. *Significant difference between Group G and group C.

Table S1: Preoperative vital signs and laboratory tests

Preoperative examination indicators	Group G	Group C	*P* value
MAP (mmHg)	92.4 ± 8.8	91.6 ± 9.1	0.690
HR (n/min)	73.2 ± 3.6	74.6 ± 3.1	0.266
T (℃)	36.4 ± 0.3	36.5 ± 0.4	0.309
Hb (g/L)	132.1 ± 11.5	136.4 ± 11.4	0.097
HCT (l/L)	39.5 ± 4.1	40.1 ± 4.8	0.549
WBC (L^{-1})	6.2 ± 1.2	5.9 ± 1.7	0.365

Abbreviation: MAP, Mean arterial pressure; HR, Heart rate; T, Temperature; Hb, hemoglobin; HCT, Hematocrit; WBC, White blood cells.

Table S2: Intraoperative fluid intake and output of patients

Input and output	Group G	Group C	*P* value
Total infusion volume (ml)	1736.7 ± 354.5	2102.5 ± 325.6*	<0.001
Crystal volume (ml)	986.6 ± 179.3	1557.2 ± 238.6*	<0.001
Colloid volume (ml)	750.1 ± 134.1	545.3 ± 101.5*	<0.001
Urine volume (ml)	312.5 ± 51.3	325.3 ± 60.7	0.312
Bleeding volume (ml)	120.8 ± 34.5	129.6 ± 38.9	0.285

*Significant difference between Group G and group C.

Table S3: MoCA score of patients at different time points

Indicators	Time points	Group G	Group C	*P* value
MoCA score	Pre-D	27.47 ± 2.15	27.12 ± 2.36	0.957
	POD_1	26.29 ± 2.56#	25.81 ± 2.68#	0.410
	POD_3	25.38 ± 2.34#	24.15 ± 2.74*#	0.044
	POD_7	26.88 ± 2.67#	25.92 ± 3.01*#	0.008

Abbreviation: MoCA, Montreal cognitive assessment; POD, day after surgery.

*Significant difference between Group G and group C.

#Significant difference compared with Pre-D.

Table S4: Postoperative complications

Complications	Group G	Group C
Total number of cases (*n*, %)	11 (27.5)	21 (52.5)*
Postoperative delirium (*n*, %)	1 (2.5)	1 (2.5)
Nausea and vomiting (*n*, %)	3 (7.5)	6 (15.0)
Abdominal distension (*n*, %)	3 (7.5)	4 (10.0)
Pulmonary infection (*n*, %)	2 (5.0)	2 (5.0)
Fever (*n*, %)	2 (5.0)	4 (10.0)
Anastomotic leakage (*n*, %)	0 (0)	1 (2.5)
Intestinal obstruction (*n*, %)	1 (2.5)	2 (5.0)

*Significant difference between Group G and group C.
